# ATAD2 interacts with C/EBPβ to promote esophageal squamous cell carcinoma metastasis via TGF-β1/Smad3 signaling

**DOI:** 10.1186/s13046-021-01905-x

**Published:** 2021-03-23

**Authors:** Lian-Jing Cao, Yi-Jun Zhang, Si-Qi Dong, Xi-Zhao Li, Xia-Ting Tong, Dong Chen, Zi-Yi Wu, Xiao-Hui Zheng, Wen-Qiong Xue, Wei-Hua Jia, Jiang-Bo Zhang

**Affiliations:** 1 State Key Laboratory of Oncology in South China, Collaborative Innovation Center for Cancer Medicine,Sun Yat-sen University Cancer Center, Guangzhou, China; 2 Department of Pathology, Sun Yat-sen University Cancer Center, Guangzhou, China; 3 Department of Urology, Sun Yat-sen University Cancer Center, Guangzhou, China

**Keywords:** Esophageal squamous cell carcinoma, Metastasis, ATAD2, TGF-β signaling pathway, C/EBPβ

## Abstract

**Background:**

Distant metastasis is the leading cause of death for esophageal squamous cell carcinoma (ESCC) with limited treatment options and unsatisfactory effectiveness. Bromodomain (BRD) containing proteins are emerging targets for cancer therapy with promising effects. As a unique member of BRD family, the function and molecular mechanism of ATAD2 in cancer development is seldomly investigated.

**Methods:**

The clinical impact of ATAD2 was assessed both at RNA and protein level in 75 and 112 ESCC patients separately. The biological function of ATAD2 was investigated in vitro and in vivo. Signaling pathway and downstream effectors of ATAD2 were identified by RNA sequencing, luciferase reporter, co-immunoprecipitation, chromatin immunoprecipitation, immunofluorescence and western blot assay.

**Results:**

We found that elevated ATAD2 expression was significantly associated with lymph node metastasis, advanced clinical stage as well as poor survival of ESCC patients. Silencing ATAD2 significantly suppressed ESCC cell migration and invasion in vitro, and inhibited tumor growth and lung metastasis in vivo. Mechanically, we identified a new cofactor, C/EBPβ. ATAD2 directly interacted with C/EBPβ and promoted its nuclear translocation, which directly bound to the promoter region of TGF-β1 and activated its expression. Further, we demonstrated that TGF-β1 activated its downstream effectors in a Smad3 dependent manner. In addition, we further found that ATAD2 promoted ESCC metastasis through TGF-β signaling induced Snail expression and the subsequent epithelial-mesenchymal transition.

**Conclusion:**

Our findings demonstrated the pro-metastatic function of ATAD2 and uncovered the new molecular mechanism by regulating C/EBPβ/TGF-β1/Smad3/Snail signaling pathway, thus providing a potential target for the treatment of ESCC metastasis.

**Supplementary Information:**

The online version contains supplementary material available at 10.1186/s13046-021-01905-x.

## Background

Esophageal cancer (EC) is one of the most common cancers occurred in the digestive system, accounting for 2.6% of all annually cancer-related deaths worldwide [1]. Esophageal squamous cell carcinoma (ESCC), accounts for over 90% of EC cases in China, is the most prevalent subtype. According to data published by American Cancer Society this year, due to the high recurrence and metastasis rate, the 5 year-survival rate of EC during 2009–2015 was only 20% [1]. Though surgery and/or chemoradiotherapy can be used as a curative intent for ESCC patients at early stage, most patients are diagnosed with advanced disease and what they confronted is less treatment options with unsatisfactory effectiveness. All of these highlight the imperative need for identification of new therapeutic targets for ESCC patients especially for those at advanced stage.

The Bromodomains (BRDs) are evolutionarily conserved protein–protein interaction domains which mainly serve as acetylated lysine residues readers and gene expression regulators [2, 3]. BRDs can regulate transcription, facilitate the assembly of larger protein complexes and chromatin modifications [3, 4]. Studies over the past years have elucidated that BRD proteins are frequently dysregulated and play an important role in tumor metastasis in esophageal cancer [5], gastric cancer [6], melanoma [7], liver cancer [8], prostate cancer [9] and glioma [10]. For example, BRD4 promoted gastric cancer metastasis through stabilization of Snail [6] and another BRD protein EP300 cooperated with c-Myc and DOT1L to derepress CDH1 transcription factors thus helping acquisition of aggressive phenotype of breast cancer [11]. Importantly, BRD is an ideal structurally target and some BRD inhibitors have been verified to be effective in various cancer types [12]. Currently, over 30 clinical trials have been carried out to evaluate the efficacy of BRD inhibitors and some of them have been proven to be effective, highlight that study of BRD proteins has far-reaching impact on cancer by hopeful clinical applications.

According to different function, BRD proteins can be classified into nine groups [3] and here, we focus on one of the BRD proteins, ATAD2. ATAD2 is a relatively new member of AAA + ATPase family, which also known as ANCCA (AAA nuclear coregulator cancer-associated protein) or TAAB (ACTR target with AAA + ATPase and bromodomain). Except for the bromodomain close to the C-terminus, ATAD2 also has two AAA + domains in the central region, which may work in concert with the BRD to exert transcriptional regulation role. In this case, it is of great important to know whether ATAD2 is overexpressed in cancer and if, upon its upregulation, ATAD2 alone or interact with other factors could work as a transcription regulator thus promoting malignant transformation and progression. Encouragingly, in cancer settings, ATAD2 was found upregulation in breast cancer for the first time and acted as a co-regulator in mediating expression of cell cycle regulators [13]. Subsequently, ATAD2 was found overexpressed in various cancer types such as prostate cancer [14], hepatocellular carcinoma [15], lung cancer, breast cancer [16], gastric cancer [17] and ovarian cancer [18] and high expression of ATAD2 was approved as an indicator of poor prognosis and had a critical part in cell proliferation, apoptosis, invasion and migration. The important function of ATAD2 in cancer attracts deep insight into the structure of ATAD2, and surprisingly, the bromodomain of ATAD2 has been validated to be a pharmacologically tractable target and a series of inhibitors are disclosing [19–21]. The function and the concrete molecular mechanism of ATAD2 in ESCC remain unclear to date.

In this study, we found that ATAD2 expression was significantly associated with lymph node metastasis and advanced clinical stage, and revealed the prognostic role of ATAD2 in a relatively large cohort of ESCC patients. Meanwhile we provide a series of experimental evidences that ATAD2 exerts a pro-metastasis role in ESCC. Mechanistic investigations suggest that ATAD2 interacts with C/EBPβ and enhances the latter’s nuclear translocation, which facilitates the transcription of TGF-β1 and the subsequent activation of TGF-β signaling pathway induced epithelial-mesenchymal transition.

## Materials and methods

### Patient samples and cell lines

Eighty-three surgically excised tumors and its adjacent non-tumor tissues were used to perform RNA isolation and assess the mRNA expression of ATAD2 and TGF-β signaling related molecules. One hundred and fifty six surgically excised tumors and 99 tumor adjacent esophageal tissues were used to evaluate ATAD2 protein expression level. All tissues were pathologically diagnosed with ESCC and collected from Sun Yat-sen University Cancer Center (SYSUCC). Prior consent from all patients or their authorized representatives and approval from the Human Ethics Approval Committee at Sun Yat-sen University Cancer Center were obtained for experimental use with human samples. GSE20347 on mRNA expression of the ESCC patients was downloaded from GEO database (https://www.ncbi.nlm.nih.gov/geo/), which consists of 17 pairs of tumor and its adjacent normal tissues. ESCC cell lines TE-1 and EC109 were purchased from the CellBank of the Chinese Academy of Sciences (Shanghai), KYSE140, KYSE510, EC9706, KYSE30 and KYSE180 were generously provided by Professor Guan from SYSUCC. All of the cell lines were cultured in RPMI-1640 media with 10% fetal bovine serum (FBS, GIBCO) and maintained in a humidified incubator under 5% CO_2_ at 37 °C.

### Antibodies and reagents

Antibodies used in this study are listed as follows: anti-ATAD2 (#ab176319, Abcam), anti-TGF-β1 (#sc-130348, Santa Cruz Biotechnology), anti-Smad2/3 (#8685, Cell Signaling Technology), anti-Smad4 (#46535, Cell Signaling Technology), anti-Phospho-Smad2 (Ser465/467)/Smad3 (Ser423/425) (#8828, Cell Signaling Technology), anti-Phospho-Smad2(Ser465/467) (#18338, Cell Signaling Technology), anti-Phospho-Smad3(Ser465/467) (#ab52903, Abcam), anti-Snail (#3879, Cell Signaling Technology), anti-E-Cadherin (#3195, Cell Signaling Technology), anti-N-Cadherin (#13116, Cell Signaling Technology), anti-Vimentin (#5761, Cell Signaling Technology), anti-β-actin (#64132, Bioworld), anti-Lamin B1 (#66095–1-Ig, Proteintech), anti-C/EBPβ (#sc-7962, Santa Cruz Biotechnology), anti-C/EBPβ (#3087, Cell Signaling Technology).

The small-molecule TGF-β1 receptor inhibitors LY2157299 and SB525334 were purchased from Selleck Biochemicals.

### Quantitative real-time PCR

Total RNA was extracted from tissues and cells using Trizol reagent (Life technologies). RNA was quantified using NanoDrop Spectrophotometer (NanoDrop Technologies) and was reverse transcribed using PrimeScript RT reagent Kit with gDNA Eraser (Takara). Quantitative real-time PCR (qRT-PCR) amplifications was performed using LightCycler 480 SYBR Green I Master (Roche) in a 20 μL reaction system containing 10 ng DNA and 250 nM of each primer; thermal cycler parameters of an LightCycler480 instrument II were 95 °C 10 min and (95 °C 15 s, 60 °C 1 min) × 45 cycles. The housekeeping gene GAPDH was used as a reference. Primers were listed in Table S1.

### Immunohistochemistry (IHC)

Tissue microarrays (TMA) constructed with the most typical areas of ESCC and normal esophageal tissues were stained with ATAD2 antibody (1: 200, ab176319, Abcam). Slides were examined by two investigators independently and those conflicting ones were re-judged to achieve consensus. Though mainly distributed in nucleus, ATAD2 protein was also detected in the cytoplasm, thus the immunoreactive score was calculated as multiplying the scores of staining intensity and percentage of ATAD2-positive cells both in cytoplasm and nucleus. Briefly, staining intensity was defined as follows: 0, negative; 1, low; 2, moderate; 3, high. The positive percentage was defined as 0, none of the cells were positively stained; 1, positively stained in 1–25% cells; 2, positively stained in 26–50% cells; 3, positively stained in 51–75% cells; 4, positively stained in 76–100% cells.

### Cell transfection and infection

Knockdown of ATAD2 in KYSE30 and KYSE510 cells was achieved using shRNA lenti-virus followed by selection using 1 μM Puromycin. The shRNA vectors were purchased from OBIO Technology including three sequences: shRNA1 GCTAGAAACATCGTTCAAA, shRNA2 GCTGCTAAGCCTCCTATAT, shRNA3 GCCTACACCCTCACTTGTT, and the control shRNA TTCTCCGAACGTGTCACGT. TE-1 and EC109 cells with ectopic expression of ATAD2 were performed by transfecting with ATAD2 expression plasmid purchased from Addgene. TGF-β response element reporter plasmid, TGF-β1 reporter plasmid and C/EBPβ expression plasmid were purchased from Public Protein/Plasmid Library. The transfection assays were performed using Lipofectamine® 3000 reagent (Thermo Scientific) according to the manufacturer’s instructions.

### Luciferase reporter assay

Luciferase reporter assay was performed as previously described [22]. Cells were seeded in a 12-well plate at a density of 1 × 10^5^ cells per well and co-transfected with indicated expression plasmids, reporter plasmids and an inner control plasmid pRL-TK. Twenty-four hours later, luciferase activity in each well was detected using the Dual-Luciferase Reporter Assay System (Promega) following the manufacturer’s protocol.

### Western blot analysis

Western blot analysis was performed as previously described [23]. Whole cell protein was extracted by RIPA lysis buffer and the ProteinExt® Mammalian Nuclear and Cytoplasmic Protein Extraction Kit (TransGene Biotech) was used to separate proteins in nuclear and cytoplasm. Lysates were quantified with BCA Protein Assay Kit (CWBio science) and separated by 8–10% sodium dodecyl sulfate-polyacrylamide gels. A polyvinylidene fluoride membrane (Millipore) was used to transfer the proteins and incubated with primary antibodies. After incubated with anti-rabbit/mouse-HRP (ZSGB-BIO) secondary antibody, specific proteins were visualized using Western Lightning ECL (Millipore).

### Co-immunoprecipitation

Cells lysates were collected and centrifuged at 4 °C. Supernatants were pre-cleared with 10 μl Protein A/G PLUS-Agarose (sc-2003, Santa Cruz) and immunoprecipitated with either 3 μg IgG or indicated antibody at 4 °C overnight with rotation. Immunoprecipitates were then pulled down with 50 μl Protein A/G PLUS-Agarose for 4 h at 4 °C with rotation. After washing for 5 times with PBS, proteins were resuspended in 30 μl SDS loading buffer and separated by SDS-PAGE gel for western blot analysis.

### Chromatin-immunoprecipitation (ChIP)-PCR assay

Cells were crosslinked with formaldehyde for 10 min at room temperature and quenched with glycine. Cells were then collected and resuspended in SDS lysis buffer with protease inhibitor cocktail. After shearing crosslinked DNA to 200-1000 bp in length, chromatin were immunoprecipitated with either 2 μg IgG or 2 μg anti-C/EBPβ antibody at 4 °C overnight with rotation. Immunoprecipitates were then pulled down with 60 μl Protein G Agarose for 1 h at 4 °C with rotation. After washing extensively (1× low salt buffer, 1× high salt buffer, 1× LiCl buffer, and 2× TE buffer) and elution, crosslinks were reversed in 5 M NaCl at 65 °C overnight. After treated with RNase A and Proteinase K, immunoprecipitated DNA was purified using spine columns and quantified.

### Cell growth and viability assays

Cells were cultivated in 96-well plates at a density of 2500 cells/well and cell viability was accessed at 24, 48, 72 and 96 h using CCK8 (Cell Counting Kit-8, DOJINDO LABORATORISE). Absorbance was quantified at 450 nm by Gen5 data analysis software. For colony forming assay, 300–1000 cells were seeded in 6-well plates and cultured for 14 days, then fixed using 100% methanol and dyed with crystal violet. Colonies with more than 50 cells were counted.

### Transwell assay

Invasion and migration assays were performed using Transwell chambers (Corning) with or without Matrigel (BD Biosciences). In brief, the upper chamber of the 24-well culture inserts were seeded with 5 × 10^4^ cells in 200 μl serum-free medium, while the lower chambers were filled with 500 μl RPMI-1640 supplemented with 20% FBS. After 16 h (KYSE30) or 36 h (KYSE510) of migration and 24 h (KYSE30) or 48 h (KYSE510) of invasion, cells remained on the upper surface of the chamber were cleaned, whereas the migrated or invaded cells were fixed in 4% paraformaldehyde and stained with crystal violet.

### In vivo assay

Five-week-old female Balb/c nude mice (Nanjing Biomedical Research Institute of Nanjing University) were maintained in specific pathogen free animal conditions. Mice were randomly divided into three groups. Group one was injected with KYSE510 cells infected with negative control shRNA (shcon), group two was injected with KYSE510 cells infected with shRNA vectors targeting ATAD2 (shATAD2) and group three was injected with KYSE510 shATAD2 cells transfected with ATAD2 plasmid (shATAD2-ATAD2). For tumorigenesis assays, 5 × 10^6^ cells in 100 μl PBS were inoculated subcutaneously into the leg region of each mouse. Tumor volume was measured every 3 days and assessed using the formula of π × (width) ^2^ × length / 6. To construct the lung metastasis model, 2 × 10^6^ cells were injected via the tail vein. Mice for xenograft model were killed at 6 weeks after injection while those for metastasis model were killed at 8 weeks. Xenograft tumors and lungs of each mouse were paraffin-embedded and stained with hematoxylin and eosin. Prior consent for animal experiments was obtained from the Animal Care and Use Committee of Sun Yat-sen University.

### Immunofluorescence (IF) staining

After 24 h seeding on the sterile slides in 24-well plates, cells were washed with Phosphate Buffered Saline (PBS) for 3 times and fixed with 4% paraformaldehyde for 20 min. Cells on the slides were permeabilized with 0.2% Triton X-100 for 5 min and blocked with 5% BSA for 1 h. Then slides were incubated with the primary antibody (1:400) overnight at 4 °C. Followed by incubation with fluorescent secondary antibody (Alexa Fluor 488 goat anti-rabbit IgG, 1:200, Cell Signaling Technology; Alexa Fluor 594 goat anti-rabbit IgG, 1:200, Cell Signaling Technology) for 1 h at room temperature without lighting, cell nuclei were stained with DAPI for 5 min. Slides were imaged by confocal microscopy (IX83, FLUOVIEW FV1200, Olympus).

### RNA sequencing

RNA-sequencing was performed using KYSE510 cells infected with two validated shRNAs targeting ATAD2 and a control shRNA by Beijing Novogene Technology Co. Ltd. Total RNA was extracted using Trizol reagent (Life technologies). Genes with fold change greater than or equal to 2 and genes with fold change less than or equal to 0.5 were considered to be significantly differentially expressed and were functional characterized using DAVID online tool (http://david.abcc.ncifcrf.gov/).

### Statistical analysis

Pearson chi-square test was used to analyze the association between ATAD2 expression and clinicopathological characteristics. Cox proportional hazards regression method was performed to predict the prognostic value of ATAD2 and other risk factors. The statistical significance between two groups was accessed by Student’s *t*-test. The differences between the two groups in cell and tumor growth curve were determined by repeated measures analysis of variance. Statistical analysis was realized by Graphpad 5 and SPSS 22 and data were shown as mean ± standard deviation (SD). *P* < 0.05 (two-sided) was considered statistically significant.

## Results

### ATAD2 is upregulated in ESCC tissues and is associated with lymph node metastasis and advanced clinical stage

**Fig. 1:**
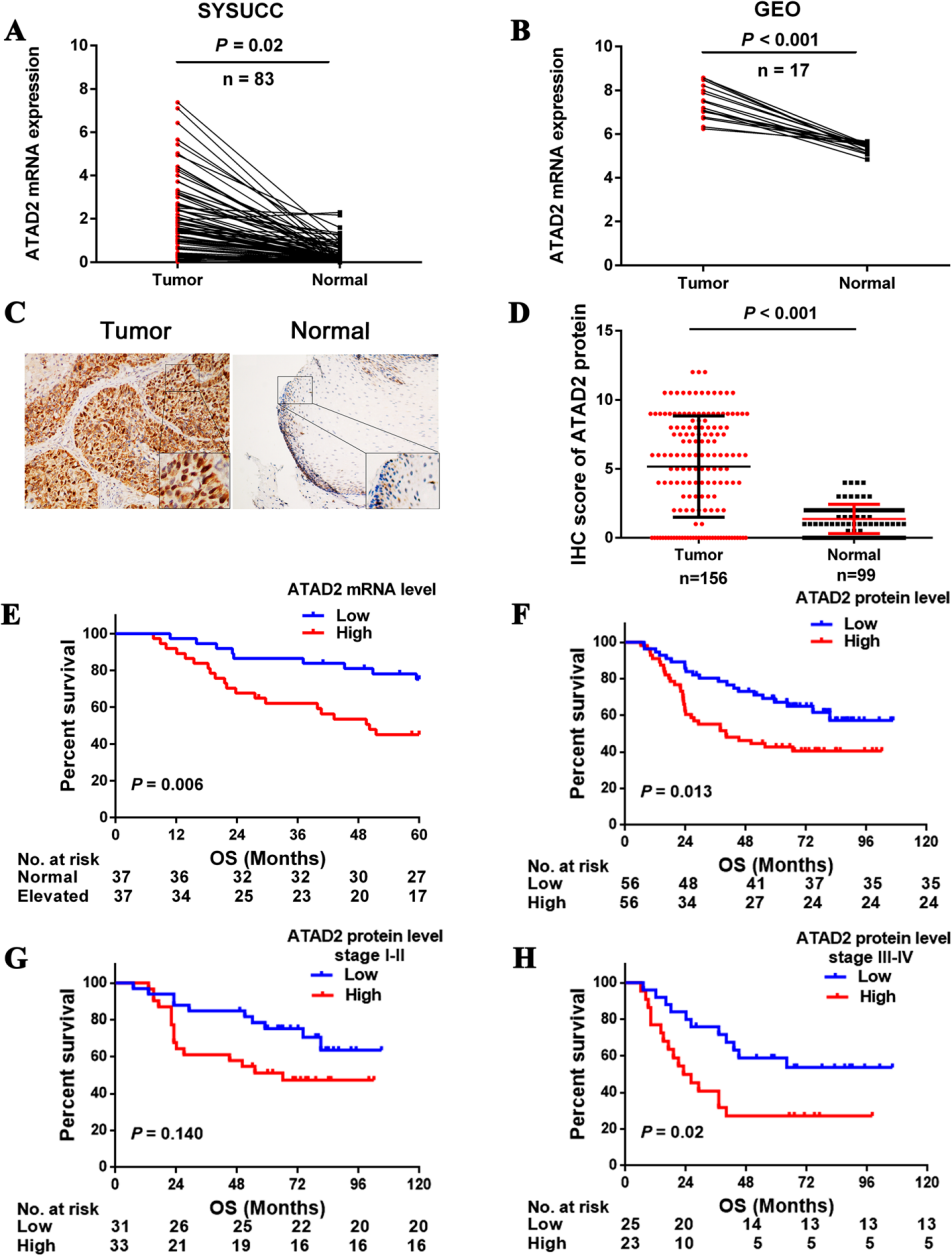
ATAD2 is upregulated and is associated with poor overall survival of ESCC patients. **a**, **b** ATAD2 mRNA expression in 83 paired primary esophageal squamous carcinoma (ESCC) and its adjacent normal tissues in SYSUCC cohort (**a**) and in GEO Database (GES20347) (**b**). **c** Representative images of positive and negative ATAD2 protein staining in paired ESCC and its adjacent normal tissues from the same patient. **d** IHC score of ATAD2 protein expression in ESCC and its adjacent normal esophageal tissues. **e**, **f** Kaplan-Meier survival curves of overall survival according to ATAD2 mRNA and protein expression (Low vs. High) in ESCC patients. **g**, **h** Kaplan-Meier survival curves of overall survival according to ATAD2 protein expression (Low vs. High) in stage I-II and stage III-IV ESCC patients

To evaluate the expression difference between ESCC and normal esophageal tissues, we assessed ATAD2 mRNA and protein expression in ESCC and normal esophageal tissues. qRT-PCR analysis detected significantly higher ATAD2 mRNA expression in cancer than normal tissues in 73 out of 83 paired tissues (Fig. 1a). We examined the GEO database and found a significant upregulation of ATAD2 mRNA expression in ESCC tissues compared with its adjacent normal tissues (GSE20347) (Fig. 1b). Consistently, IHC results of TMA showed that ATAD2 protein was elevated in 67.3% (105/156) of the ESCC tissues, which was dramatically increased compared with only 10.1% (10/99) in normal esophageal tissues (Fig. 1c, d). We then analyzed the correlation between ATAD2 expression and clinicopathological features, and ATAD2 expression was found to be significantly associated with lymph node metastasis (*P* = 0.002) and advanced stage (*P* = 0.028) (Table 1). Taken together, ATAD2 is significantly upregulated and correlated with lymph node metastasis, suggesting a potential oncogenic and pro-metastasis role in ESCC.

**Table 1:** The association between ATAD2 expression and the clinicopathological variables

Variables	Cases	ATAD2 Expression		***P***
		Low (%)	High (%)	
**Gender**				0.514
Male	50	24 (48.0)	26 (52.0)	
Female	25	14 (56.0)	11 (44.0)	
**Age (years)**				0.134
< 57	39	23 (59.9)	16 (40.1)	
≥ 57	36	15 (41.7)	21 (58.3)	
**Tumor Location**				0.175
Upper	9	4 (44.4)	5 (55.6)	
Middle	49	22 (44.9)	27 (55.1)	
Lower	17	12 (70.6)	5 (29.4)	
**Histologic Grade**				0.624
G1	22	13 (59.1)	9 (40.9)	
G2	33	16 (48.5)	17 (51.5)	
G3	20	9 (45.0)	11 (55.0)	
**T Status**				0.054
T1–T2	19	6 (31.6)	13 (68.4)	
T3–T4	56	32 (57.1)	24 (42.9)	
**Lymph Node Metastasis**				**0.002**
N0	42	28 (66.7)	14 (33.3)	
N1,2,3	33	10 (30.3)	23 (69.7)	
**TNM Stage**				**0.028**
I–II	36	23 (63.9)	13 (36.1)	
III–IV	39	15 (38.5)	24 (61.5)	
**Smoking**				0.253
No	25	15 (60.0)	10 (40.0)	
Yes	50	23 (46.0)	27 (54.0)	
**Alcohol**				0.423
No	44	24 (54.5)	20 (45.5)	
Yes	31	14 (45.2)	17 (54.8)	

*P* < 0.05 in bold

### ATAD2 is an independent predictor of poor survival in ESCC patients

We performed Kaplan–Meier analysis to identify the prognostic value of ATAD2 both at mRNA and protein level. High expression of ATAD2 mRNA and protein were both significantly associated with poor overall survival in ESCC patients (Fig. 1e, f). Univariate Cox regression analysis showed that high expression of ATAD2 (hazard ratio [HR] 1.63, *P* = 0.015) along with advanced clinical stage and poor histological differentiation were significantly related to poor overall survival in ESCC patients (Table S2). Further multivariate analysis after adjusted the potential confounding factors demonstrated that ATAD2 overexpression was an independent prognostic marker for ESCC patients (HR 2.44, *P* = 0.003) (Table S2). When patients were further divided into subgroups according to clinical stage (I-II vs. III-IV), a significant association was found between ATAD2 expression and overall survival in stage III-IV patients (*P* = 0.02) (Fig. 1h), while no significant association was found between ATAD2 expression and survival in stage I-II patients (*P* = 0.140) (Fig. 1g).

### ATAD2 promotes ESCC cells proliferation and tumorigenesis

**Fig. 2:**
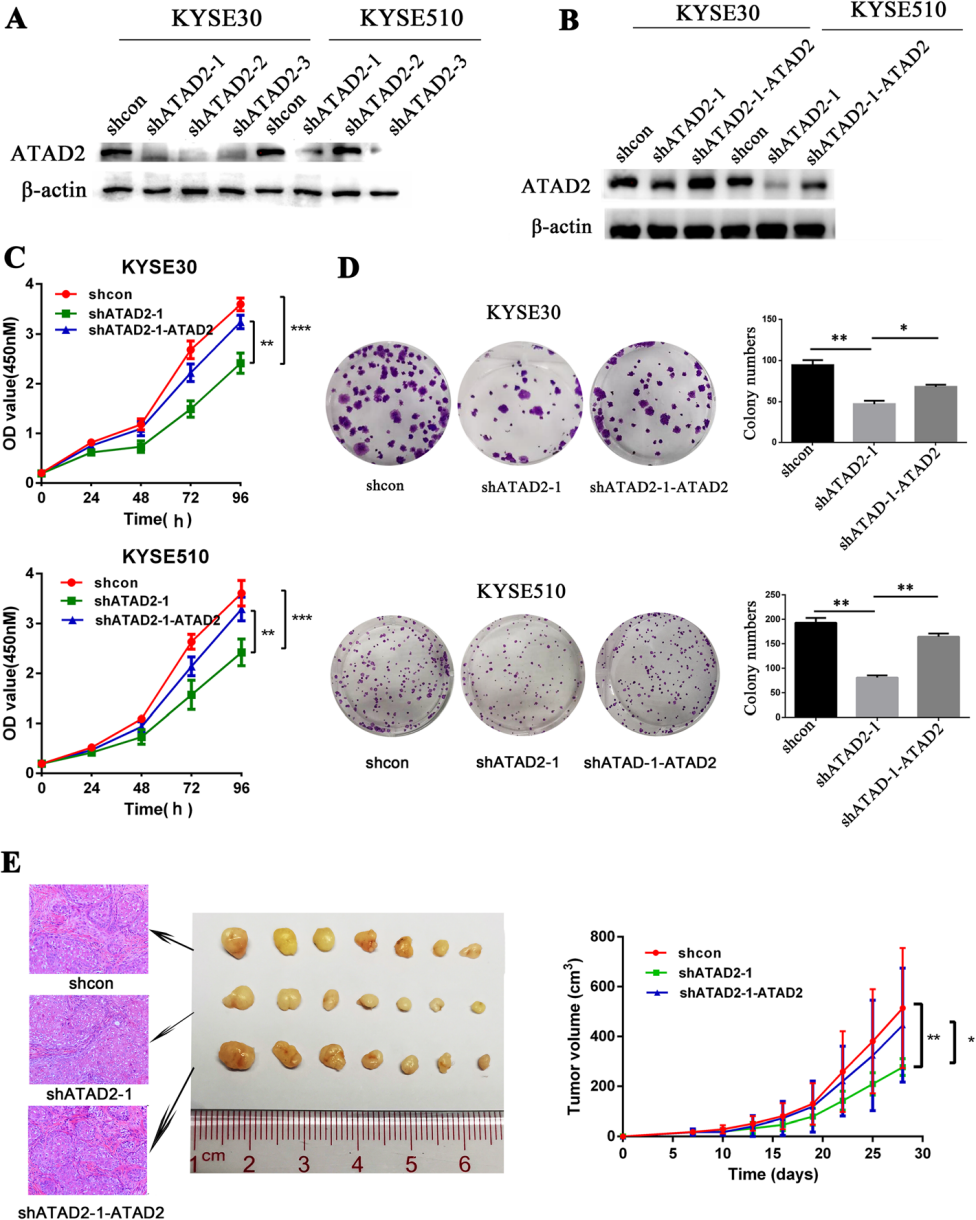
ATAD2 promotes ESCC cell proliferation in vitro and tumorigenesis in vivo. **a**, **b** Knockdown of ATAD2 by three shRNAs and restoration of ATAD2 expression in KYSE30 and KYSE510 cells were confirmed by western blot analysis. The following functional experiments in KYSE30 and KYSE510 cells are all grouped in accordance with (**b**). **c** Effect of altered ATAD2 expression on cell growth by CCK8 assay. **d** Effect of altered ATAD2 expression on cell colony formation and colonies of each group were quantified. **e** Effect of altered ATAD2 expression in KYSE510 cells on tumor growth in nude mice (*n* = 7 tumors per group), tumor size was measured every 3 days after 1 week after injection. Tumor tissues were confirmed by hematoxylin-eosin staining. Data was presented as mean ± standard deviation (SD) (*P < 0.05; **P < 0.01; *** *P* < 0.001)

Three lenti-virus shRNAs targeting ATAD2 and a control shRNA were used to infect KYSE510 and KYSE30 cells which showed relatively high expression of ATAD2. In addition, we restored ATAD2 expression in ATAD2 knockdown KYSE510 and KYSE30 cells to affirm the function of ATAD2. Western blot analysis showed a near-complete loss of ATAD2 in two of the three shRNAs (Fig. 2a) and a significantly overexpression when transfected with ATAD2 plasmid (Fig. 2b).

We conducted CCK-8 assay and colony formation assay. The results showed that knockdown of ATAD2 significantly attenuated cell growth and colony formation ability in KYSE510 and KYSE30 cells compared to control cells, however, when ATAD2 expression was restored, the ATAD2-induced effects on cell proliferation and survival were all partially reversed (Fig. 2c, d). To further confirm the function of ATAD2 on growth characteristics, we performed the xenograft model using KYSE510 cells infected with shcon, shATAD2–1 and KYSE510 shATAD2–1 cells with ectopic ATAD2 expression. Consistent with in vitro assays, tumor growth was significantly decreased in ATAD2 knockdown group compared with control group (*P* < 0.01), and the group with ectopic expression of ATAD2 grew larger tumors compared with the ATAD2 knockdown group (*P* < 0.05) (Fig. 2e).

### ATAD2 promotes metastasis of ESCC cells in vitro and in vivo

**Fig. 3:**
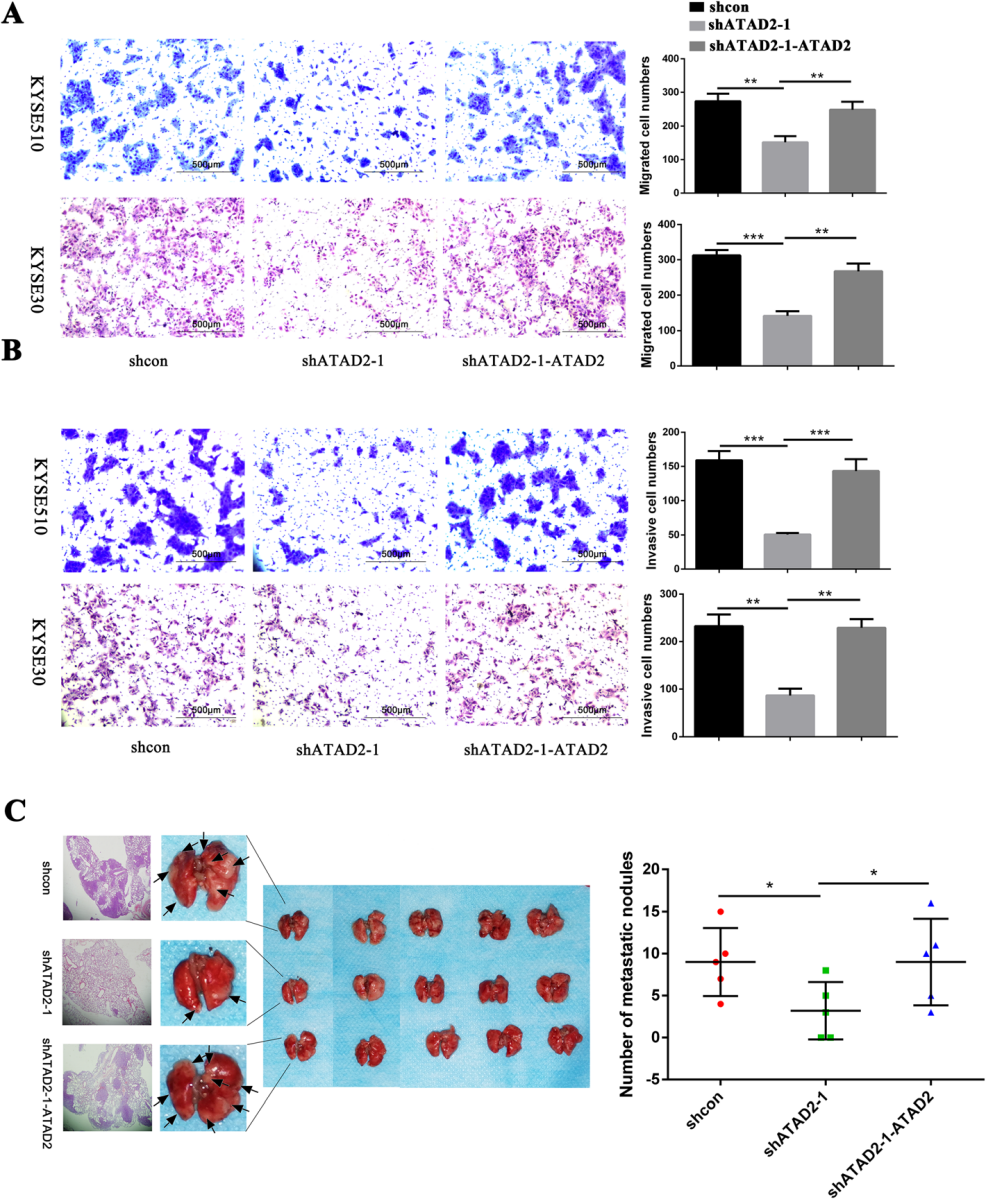
ATAD2 promotes ESCC cell migration, invasion in vitro and metastasis in vivo*.* **a**, **b** Effect of altered ATAD2 expression on cell migration and invasion ability using transwell assay. The migrated and invasive cells were counted and analyzed. **c** Macroscopic appearances of lung images of each group, black arrows indicate the tumor nodules. Lung metastasis nodules were confirmed by hematoxylin-eosin staining. Number of metastatic nodules in each group was counted and analyzed. Data was analyzed using unpaired student’s *t*-test and presented as mean ± SD (*P < 0.05; **P < 0.01, *** *P* < 0.001)

We then investigated the effect of ATAD2 on tumor metastasis through in vitro transwell assay and in vivo murine lung metastatic model. Transwell assay showed knockdown of ATAD2 dramatically reduced cell migration and invasion ability both in KYSE510 and KYSE30 cells while these effects were reversed when ATAD2 expression was restored (Fig. 3a, b, S1A). To validate the effect of ATAD2 on cell metastasis in vivo, we constructed murine lung metastatic model and the experimental groups were the same as the xenograft assay. The lung metastatic nodules were significantly decreased in mice injected with ATAD2 knockdown cells compared with the control group (*P* < 0.05) and mice injected with ATAD2 restored cells grew significantly more lung metastatic nodules than the ATAD2 knockdown group (*P* < 0.05) (Fig. 3c).

### ATAD2 activates TGF-β signaling pathway

**Fig. 4:**
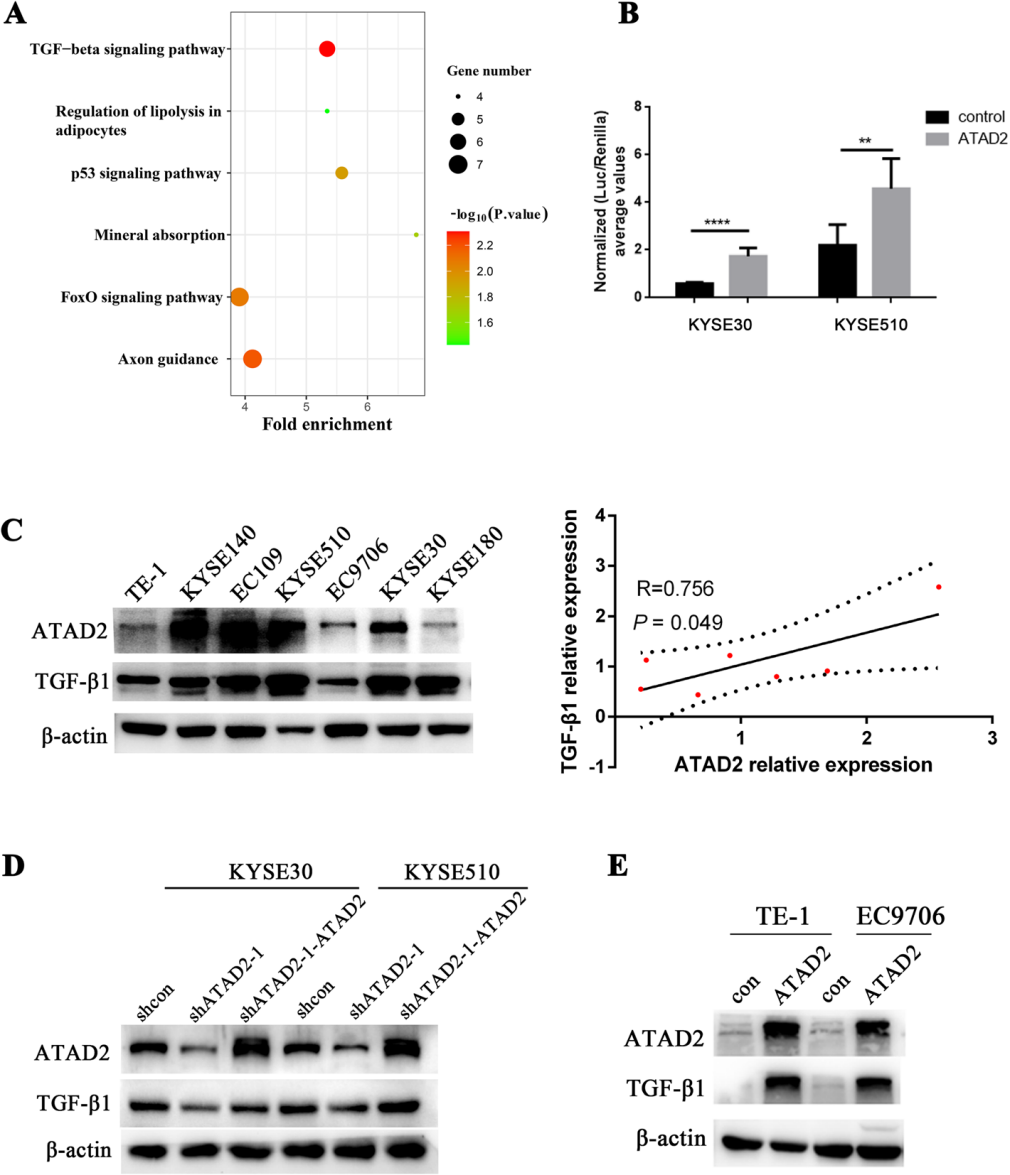
ATAD2 activates TGF-β signaling pathway and TGF-β1 gene expression. **a** KEGG pathway analysis using significantly differentially expressed genes between ATAD2 knockdown KYSE510 cells and control cells based on RNA-sequencing data. **b** TGF-β response element luciferase activity induced by ATAD2 in KYSE30 and KYSE 510 cells. **c** ATAD2 and TGF-β1 expression in a panel of ESCC cell lines analyzed by western blot. Expression of ATAD2 and TGF-β1 protein were quantified and analyzed using Pearson correlation method. **d** Effect of ATAD2 knockdown and restoration on TGF-β1 analyzed by western blot. **e** Effect of ATAD2 overexpression on TGF-β1 analyzed by western blot

To elucidate the molecular mechanism of ATAD2 in promoting tumor metastasis in ESCC, we performed RNA sequencing on KYSE510 cells infected with two validated shRNAs targeting ATAD2 and a control shRNA. We integrated the significant different expression genes of shATAD2–1 vs. shcon and shATAD2–3 vs. shcon, and obtained 395 overlapping genes. In order to identify pathways regulated by ATAD2, the KEGG pathway enrichment analysis of the overlapping genes was performed using the online tool DAVID v6.7. TGF-β signaling pathway listed on the top of all the biological pathways enriched with *P* < 0.05 (Fig. 4a, Table S3). To confirm the regulatory role of ATAD2 on TGF-β signaling pathway, several methods were applied. TGF-β response element luciferase reporter assay showed that ectopic expression of ATAD2 dramatically increased the luciferase activity of TGF-β signaling reporter both in KYSE510 and KYSE30 cells (Fig. 4b). Additionally, we detected TGF-β1 protein expression in the aforementioned 7 ESCC cell lines. ATAD2 and TGF-β1 protein expression were significantly positively correlated by correlation analysis (Fig. 4c). Moreover, we also validated TGF-β1 expression in ATAD2 knockdown and overexpression cells. TGF-β1 was near-completely lost when ATAD2 was knockdown and was upregulated when ATAD2 was reversed in KYSE510 and KYSE30 cells (Fig. 4d), while overexpressing ATAD2 in TE-1 and EC9706 cells with relatively low ATAD2 expression induced evidently enhanced TGF-β1 expression (Fig. 4e).

### ATAD2 promotes ESCC metastasis through TGF-β signaling induced epithelial-mesenchymal transition

**Fig. 5:**
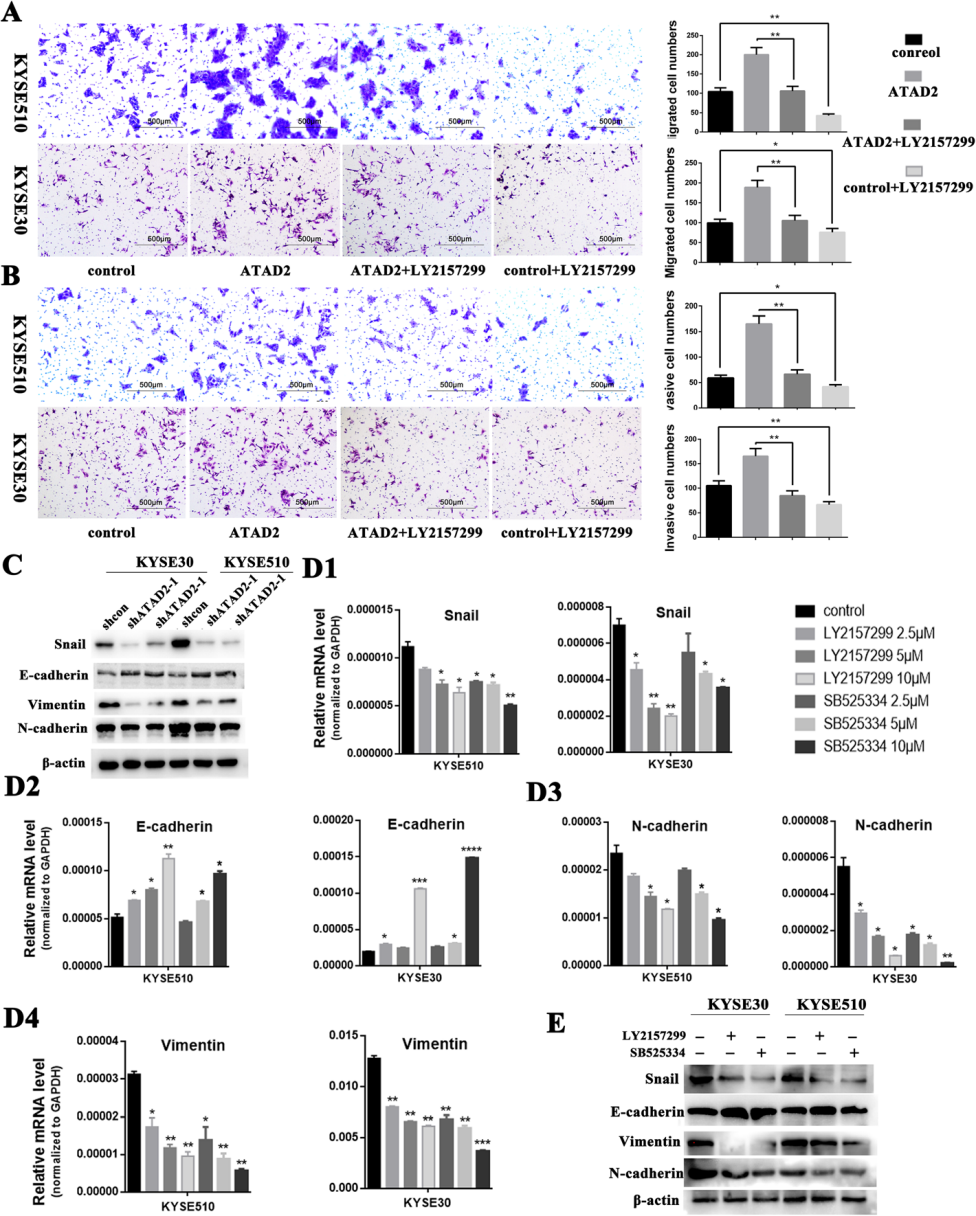
ATAD2 exerts pro-metastatic function depending on the TGF-β signaling induced epithelial-mesenchymal transition. **A**, **B** Effect of TGF-β1 receptor inhibition on KYSE510 and KYSE30 cell migration and invasion ability using transwell assay. TGF-β1 receptor was inhibited with 5 μM LY2157299. The migrated and invasive cells were counted and analyzed. **C** Effect of ATAD2 knockdown on transcription factor Snail, epithelial marker E-cadherin and mesenchymal markers Vimentin and N-cadherin analyzed by western blot. **D1-4**, **E** Effect of TGF-β1 receptor inhibition on transcription factor Snail, epithelial marker E-cadherin and mesenchymal markers Vimentin and N-cadherin analyzed by qRT-PCR (**D1-4**) and western blot (**E**) respectively. For qRT-PCR, TGF-β1 receptor was inhibited with 2.5 μM, 5 μM, 10 μM LY2157299 and SB525334 for 24 h, and 5 μM LY2157299 and SB525334 were applied for 48 h for western blot analysis. Data was presented as mean ± SD (*P < 0.05; **P < 0.01, *** *P* < 0.001)

To elucidate the role of TGF-β signaling in ATAD2-mediated tumor metastasis, we blocked TGF-β signaling in ATAD2-overexpressed KYSE510 and KYSE30 cells using TGF-β1 receptor inhibitors. As shown in Fig. 5A and B, ectopic expression of ATAD2 significantly promoted KYSE510 and KYSE30 cells migration and invasion, whereas they were significantly inhibited after TGF-β1 receptor blocking, inferring that the pro- metastatic effect of ATAD2 is at least in part depending on TGF-β signaling in ESCC. Meanwhile, after knocking down of ATAD2 expression, western blot analysis showed a significantly upregulation of epithelial marker E-cadherin as well as down-regulation of mesenchymal markers (Snail, Vimentin and N-cadherin) both in KYSE510 and KYSE30 cells (Fig. 5C). In addition, blocking of TGF-β1 receptor using TGF-β1 receptor inhibitors also led to the upregulation of epithelial marker and down-regulation of mesenchymal markers in KYSE510 and KYSE30 cells both at RNA and protein levels (Fig. 5D, E). These results collectively indicate that the pro-metastatic ability of ATAD2 is, to a large extent, depending on TGF-β signaling induced epithelial-mesenchymal transition.

### ATAD2 functions through TGF-β1/Smad3 pathway

**Fig. 6:**
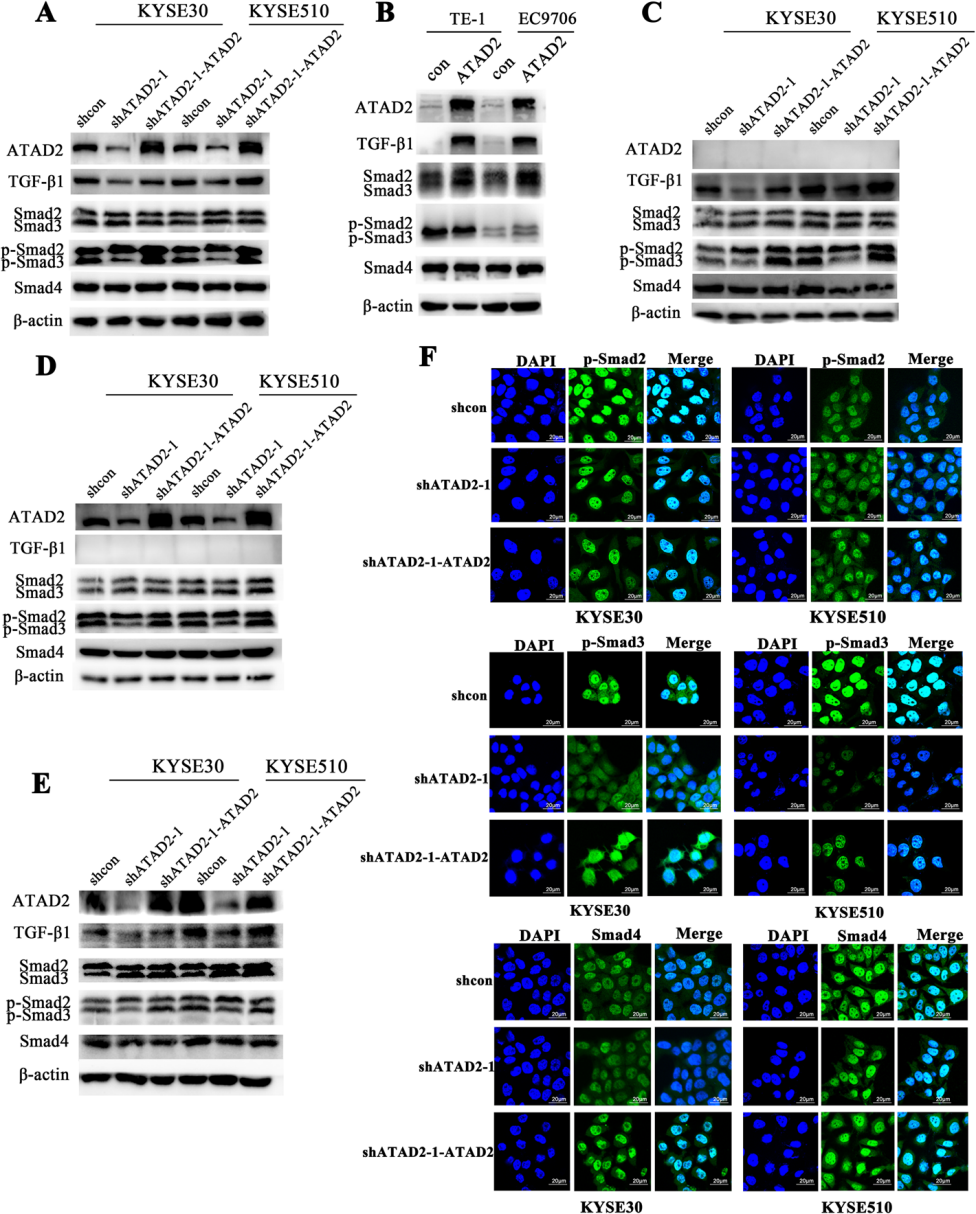
ATAD2 regulates TGF-β1/Smad3 pathway which is independent of Smad2 and Smad4. **a** Effect of ATAD2 knockdown and restoration on expression of TGF-β1 and its downstream effectors analyzed by western blot in KYSE30 and KYSE510 cells using the whole cell extracts. **b** Effect of ATAD2 overexpression on TGF-β1 and its downstream effectors analyzed by western blot in TE-1 and EC9706 cells using the whole cell extracts. **c**, **d** Effect of ATAD2 knockdown and restoration on TGF-β1 and its downstream effectors analyzed by Western blot in KYSE30 and KYSE510 using cytoplasmic and nuclear proteins separately. **e** Effect of ATAD2 knockdown and restoration on TGF-β1 and its downstream effectors in xenograft tumors. **f-h** Effects of ATAD2 knockdown and restoration on localization of p-Smad2 (green) (**f**), p-Smad3 (green) (**g**), Smad4 (green) (**h**) and DAPI (blue) performed by immunofluorescence staining

The canonical TGF-β1 pathway functioned as follows: binding of TGF-β1 to its receptors leading to phosphorylation of Smad2 (p-Smad2) and Smad3 (p-Smad3), p-Smad2 and p-Smad3 form into trimeric complexes with Smad4, then the trimer complexes translocate to nucleus and regulate the expression of a subset of genes. Thus we attempted to find out whether ATAD2 worked through the canonical TGF-β1/Smad pathway. We first performed western blot using protein extracted from the whole cells. The results demonstrated that in addition to TGF-β1, only p-Smad3 was downregulated in ATAD2 knockdown cells and upregulated when ATAD2 expression was restored (Fig. 6a). Similarly, overexpression of ATAD2 resulted in increased expression of TGF-β1 and enhanced activation of Smad3 (Fig. 6b, g). Phosphorylation of Smad2 did not change concomitant with TGF-β1 (Fig. 6f). We then separated proteins in nuclear and cytoplasm to further investigate the downstream effectors of TGF-β1 regulated by ATAD2 in ESCC. Only activation of Smad3 was changed in accordance with altered expression of ATAD2 both in cytoplasm (Fig. 6c) and nuclear (Fig. 6d). Intriguingly, decreased activation of Smad3 induced by ATAD2 knockdown did not affect the nuclear translocation of Smad4 (Fig. 6c, h). To further consolidate the mechanism, we performed western blot analysis using proteins extracted from the xenograft tumors of each group and obtained the same results (Fig. 6e). These results demonstrate that ATAD2 potentiate TGF-β1 signaling pathway in a Smad3-dependent manner which is independent of Smad2 and Smad4.

### C/EBPβ direcctly binds to the promoter region of TGF-β1 and activates its transcription

**Fig. 7:**
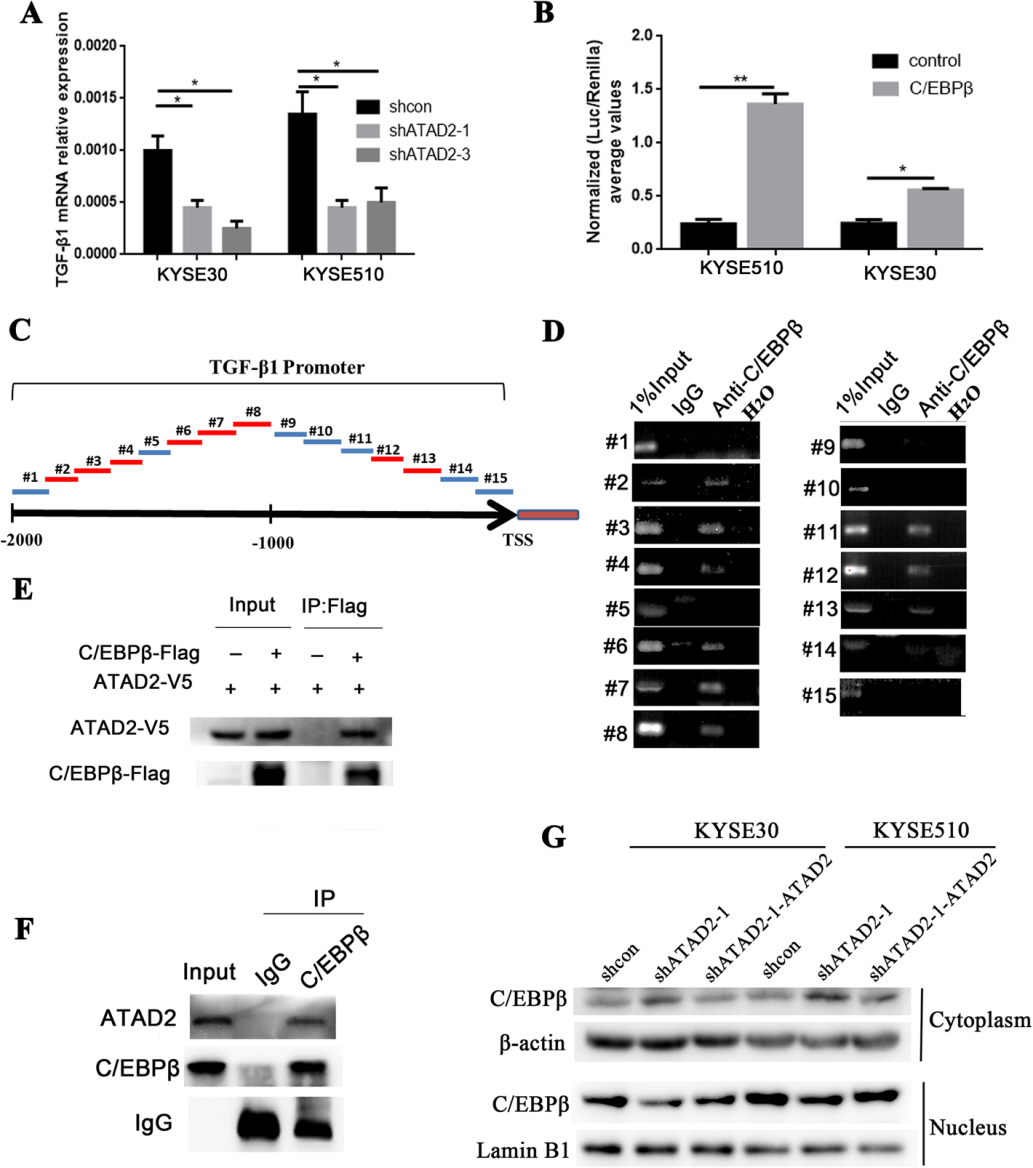
ATAD2 facilitates nuclear translocation of C/EBPβ which directly activates TGF-β1 gene transcription. **a** Effect of ATAD2 knockdown on TGF-β1 mRNA expression in KYSE30 and KYSE510 cells by qRT-PCR. **b** TGF-β1 luciferase activity induced by C/EBPβ in KYSE510 and KYSE30 cells. **c** Schematic figure summarizing ChIP-PCR primer sets in TGF-β1 promoter region. **d** C/EBPβ directly bound to the TGF-β1 promoter regions (region #2–4, #6–8, and #11–13). **e** The interaction between ATAD2 and C/EBPβ was verified by co-immunoprecipitation. Anti-Flag antibody was used to immunoprecipitate Flag-tagged C/EBPβ from whole cell extracts prepared from HEK293T cells co-expressing V5-ATAD2 and Flag-C/EBPβ. **f** Endogenous interaction of ATAD2 and C/EBPβ in KYSE510 cells verified by co-immunoprecipitation. **g** Effect of ATAD2 knockdown on C/EBPβ expression by western blot in KYSE30 and KYSE510 cells using cytoplasmic and nuclear proteins separately. Data was presented as mean ± SD (*P < 0.05; **P < 0.01)

To identify whether regulation of ATAD2 on TGF-β1 expression is at the transcriptional or translational level, qRT-PCR was performed. PCR analysis demonstrated a significantly downregulation of TGF-β1 mRNA expression in ATAD2 knockdown KYSE510 and KYSE30 cells (Fig. 7a). Given ATAD2 is not a transcriptional factor and lacks of the ability to directly activate TGF-β1 transcription, we hypothesized that ATAD2 works as a transcription coactivator to facilitate TGF-β1 transcription. The online transcription factors prediction database PROMO (http://alggen.lsi.upc.es/cgi-bin/promo_v3/promo/promoinit.cgi?dirDB=TF_8.3) was used to predicted the transcription factors of TGF-β1 gene (Figure S1B) and according to the prediction results, we examined the transcriptional activation of TGF-β1 by C/EBPβ, YY1 and c-Myc. Luciferase reporter assay showed that only C/EBPβ significantly enhanced TGF-β1 transcription in KYSE510 and KYSE30 cells (Fig. 7b, S1C, D). We then investigated whether C/EBPβ directly induced TGF-β1 transcription. Chromatin immunoprecipitation-PCR assay (ChIP-PCR) was performed by using 15 sets of primers coving the promoter region of TGF-β1 gene from − 2000 to + 50 (Fig. 7c). The result showed that C/EBPβ could directly bind to the multiple sites of TGF-β1 promoter region (Fig. 7d). Our results revealed C/EBPβ as a transcription activator of TGF-β1 gene.

### ATAD2 interacts with C/EBPβ and facilitates its nuclear translocation

To investigate the interaction between ATAD2 and C/EBPβ, we first expressed V5-tagged ATAD2 and Flag-tagged C/EBPβ in HEK293T cells. The result of co-immunoprecipitation demonstrated the interaction of ATAD2 with C/EBPβ (Fig. 7e). Furthermore, endogenous reciprocal co-immunoprecipitation in KYSE510 cells using anti-C/EBPβ antibody verified that C/EBPβ had a strong binding to endogenous ATAD2 (Fig. 7f). Since neither whole C/EBPβ mRNA nor protein expression was altered in ATAD2 knockdown KYSE510 and KYSE30 cells (Figure S1E, F), we separated proteins in nuclear and cytoplasm to detect the nuclear translocation alteration of C/EBPβ. Knockdown of ATAD2 resluted in a upregulation of cytoplasmic level of C/EBPβ and downregualtion of nuclear level of C/EBPβ. And when ATAD2 expression was reversed, nuclear expression of C/EBPβ was upregualted and cytoplasmic expression of C/EBPβ was downregulated (Fig. 7g). Immunofluorescence staining of C/EBPβ obtained the consistent results (Figure S1G). Collectively, these results suggest that ATAD2 acts as an interaction partner of C/EBPβ and promoted the nuclear translocation of C/EBPβ.

### ATAD2 is positively correlated with TGF-β1 signaling

**Fig. 8:**
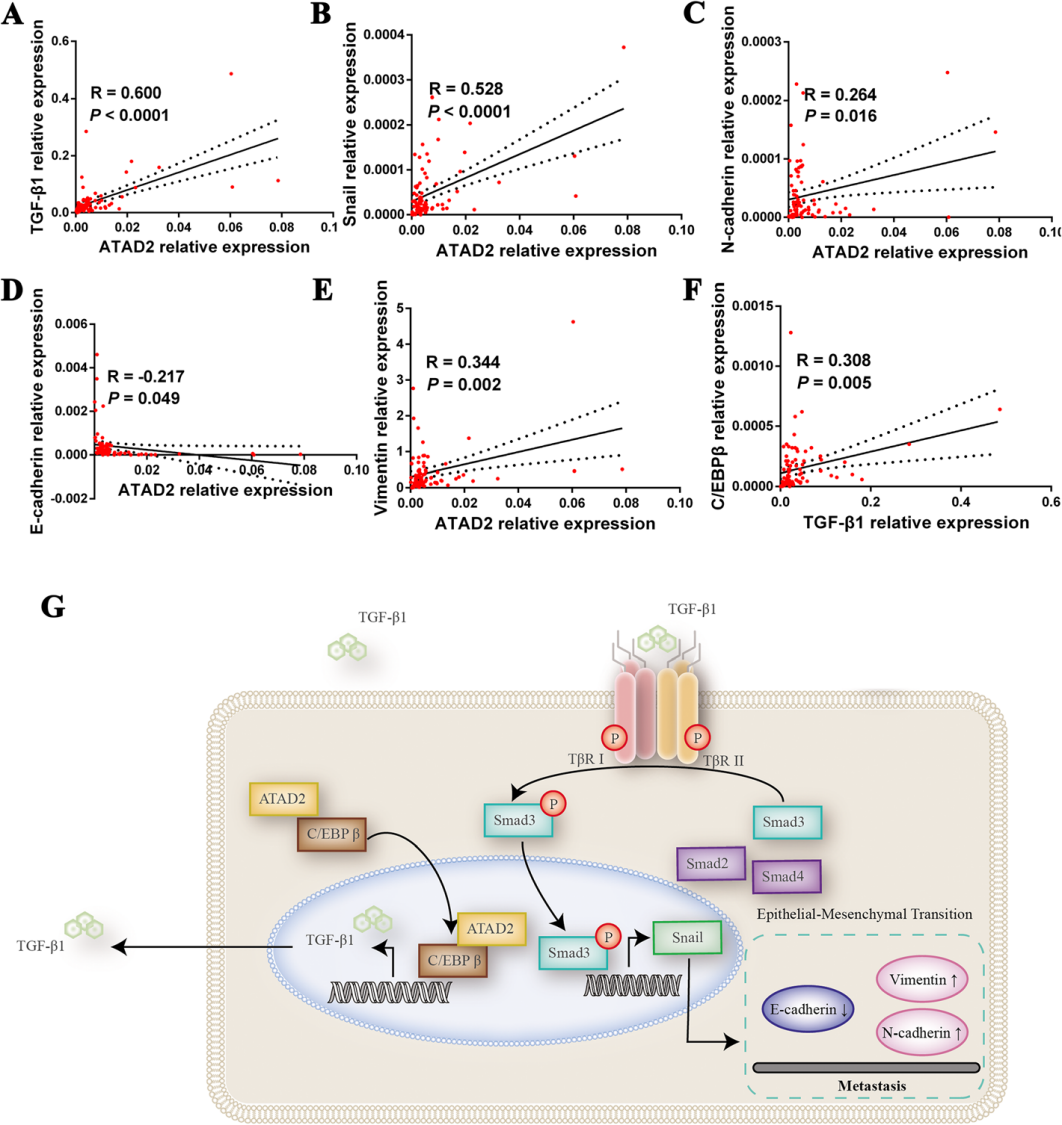
ATAD2 is positively correlated with TGF-β1 signaling pathway in ESCC patients. **a**-**e** Correlation analysis of ATAD2 with TGF-β1 and epithelial-mesenchymal transition markers in ESCC patients. **f** Correlation analysis of expression of TGF-β1 and C/EBPβ in ESCC patients. **g** Mechanistic model of oncogenic function of ATAD2 in ESCC. ATAD2 interacts with C/EBPβ and enhances the latter’s nuclear translocation. ATAD2 interacts with C/EBPβ and enhances the latter’s nuclear translocation. C/EBPβ directly binds to the promoter region of TGF-β1 gene and facilitates its transcription and the subsequent activation of TGF-β signaling pathway induced epithelial-mesenchymal transition

To provide clinical relevance of ATAD2 and TGF-β1 signaling, we detected mRNA expression of C/EBPβ, TGF-β1, Snail, E-cadherin, N-cadherin and Vimentin in 83 ESCC tissues and found that ATAD2 was significantly positively correlated with TGFβ1, Snail, N-cadherin and Vimentin and negatively correlated with E-cadherin (Fig. 8a-e). In addition, there was a significantly positively association between TGFβ1 and C/EBPβ (Fig. 8f). These data further validated the correlations between ATAD2 and TGF-β1 and epithelial-mesenchymal transition as well as the correlation between C/EBPβ and TGF-β1 in ESCC patients.

## Discussion

ATAD2 is a bromodomain containing protein which might play a vital role in cell malignant transformation and metastasis. However, the oncogenic role and molecular mechanism of ATAD2 in ESCC metastasis remains unclear. Here, we demonstrated that ATAD2 mRNA and protein expression were both upregulated in primary ESCC tissues. High level of ATAD2 expression was correlated with lymph node metastasis and advanced clinical stage and predicted poor overall survival in ESCC patients, which indicated an oncogenic role of ATAD2 in ESCC. Consistent with our hypothesis, functional experiments revealed that ATAD2 played a critical part in cell proliferation and metastasis in ESCC both in vitro and in vivo. Mechanically, through RNA-sequencing, our study uncovered the association between ATAD2 and TGF-β signaling pathway for the first time and further demonstrated that the pro-metastatic ability of ATAD2 is, to a large extent, depending on TGF-β signaling induced epithelial-mesenchymal transition. Importantly, we illustrated that ATAD2 worked as a novel cofactor of C/EBPβ to enhance its nuclear translocation and facilitate TGF-β signaling pathway (Fig. 8g).

Since the initial symptom is not obvious, ESCC patients are often diagnosed at advanced stage. Surgery as a curative treatment is only suitable for those with early stage. Treatment is less and median survival is only around 1 year for those majorities with metastatic or unresectable ESCC [24–26]. New targeted therapy is in desperate need to achieve better outcome in advanced ESCC patients. In this regard, we examined the association between ATAD2 expression and patients outcome and discovered that high level of ATAD2 was significantly correlated with poor overall survival of ESCC patients especially those with advanced stage. Further multivariate Cox regression model showed that high expression of ATAD2 was an independent prognostic marker for advanced ESCC patients, which suggested that ATAD2 may be used as a hopeful therapeutic target.

The Bromodomains are evolutionarily conserved modules which mainly recognize acetylated Lys residues and thereby function in gene regulation. Notably, most BRD proteins also contain other conserved domains which function independently or cooperate with BRD functioning in protein-protein or protein-nucleic acid interactions [3]. ATAD2 contains a bromodomain close to the C-terminus and two AAA + domains in the central region. ATAD2 has previously been demonstrated to function as transcriptional co-regulators of estrogen receptor-α [13] and MYC [27]. It was shown that ATAD2 worked as a scaffold which recruited E2F transcription factors thus promoting gene transcription [28]. Other BRD proteins were also reported to regulate transcription by serving as scaffolds that dominant the recruitment of transcriptional factors to chromatin [4]. Our data showed that ATAD2 interacted with C/EBPβ and promoted C/EBPβ nuclear translocation. C/EBPβ, a member of the C/EBP Leucine zipper transcription factor family, could directly bind to the promoter region of TGF-β1 gene and activated its transcription and the downstream signaling pathway. Our results demonstrated a new interaction transcription factor of ATAD2, providing important reference for exploring the molecular mechanism of ATAD2 in other cancer types.

TGF-β signaling pathway is reported to have dual function in various cancer types including ESCC, acting as a tumor promoter or tumor suppressor depending on tumor stage and context [29–31]. Several studies have devoted to investigate the role of TGF-β signaling pathway in ESCC. The majority demonstrated the tumor-promoting role of TGF-β signaling pathway in ESCC [32–34], while several studies draw the contrary conclusion [35, 36]. We discovered TGF-β signaling pathway listed on the top one of the pathways associated with ATAD2 via RNA-sequencing and proved the regulation role of ATAD2 on TGF-β1, which clarified a tumor-promoting role of TGF-β signaling pathway in ESCC. As ATAD2 was significantly associated with lymph node metastasis in ESCC patients and promoted cell migration and invasion as well as lung metastasis in mice, we further devoted to find out the underline pro-metastatic mechanism of ATAD2. Epithelial-mesenchymal transition is considered as the main mechanism of cancer metastasis. Specifically, tumor cells utilize the transition to facilitate the process of migration, invasion and the final plantation [37]. We found ATAD2 positively regulated epithelial-mesenchymal transition in ESCC cells through upregulation of Snail, Vimentin and N-cadherin and downregulation of E-cadherin and further indicated that the pro-metastatic ability of ATAD2 is, to a large extent, depending on TGF-β signaling induced epithelial-mesenchymal transition. Since we also found that ATAD2 was significantly correlated with lymph node metastasis and advanced clinical stage in ESCC patients, and TGF-β signaling pathway was proved to promote tumor progression in advanced-stage tumors, thus we hypothesize that function of TGF-β signaling pathway regulated by ATAD2 may mainly work in advanced ESCC patients. More studies are needed to elucidate it.

The TGF-β signaling pathway functioned canonically in a Smads dependent manner including Smad2, Smad3 and Smad4. However, our study found the TGF-β signaling pathway regulated by ATAD2 in ESCC worked through Smad3 but independent of Smad2 and Smad4. Consistent with our study, a recent study in esophageal cancer also demonstrated that TGF-β signaling pathway functioned in a Smad4-independent way [38]. More studies in recent years have reported that TGF-β mediated Smad3 rather than Smad2 played a crucial role in cancer [39, 40]. Additionally, Smad3 was capable of binding directly to DNA to regulate gene expression [31], which was in step with our finding that it was Smad3 rather than the Smads complex that bound to DNA to regulate expression of a subset of genes in ESCC.

## Conclusions

Our data uncovered for the first time that ATAD2 interacted with C/EBPβ and promoted metastasis via C/EBPβ/TGF-β1/Smad3/Snail signaling pathway. This observation provides a new molecular mechanism of ATAD2 in ESCC. Based on our study, we propose that ATAD2 is a qualified prognostic marker and serves as a promising therapeutic target for ESCC patients especially for those with metastasis or unresectable tumors.

## Supplementary Information

**Additional file 1: Table S1.** Primers used for quantitative real-time PCR.

**Additional file 2: Table S2.** Univariate and multivariate analyses of overall survival in all patients.

**Additional file 3: Table S3.** KEGG pathways of overlapping genes from DAVID analysis.

**Additional file 4:** **Supplemental Figure 1.** (A) ATAD2 overexpression, knockdown, and restoration in KYSE30 and KYSE510 cells were confirmed by western blot analysis. (B) Predicted transcription factors of TGF-β1 gene obtained from PROMO dataset. (C, D) TGF-β1 luciferase activity induced by YY1 (C) and c-MYC (D) in KYSE510 and KYSE30 cells. (E, F) Effect of ATAD2 knockdown on C/EBPβ mRNA (E) and protein expression (F) in KYSE30 and KYSE510 cells were validated by qRT-PCR and western blot respectively. (G) Effects of ATAD2 knockdown and restoration on localization of C/EBPβ (green) in KYSE30 and KYSE510 cells performed by Immunofluorescence staining. Data was presented as mean ± SD. (ns: non-significant difference was detected).

## Data Availability

The datasets generated and/or analysed during the current study are available in the Reserch Data Deposit repository, www.researchdata.org.cn.

## Abbreviations

ESCC: Esophageal squamous cell carcinoma
BRD: Bromodomain
EC: Esophageal cancer
qRT-PCR: Quantitative real-time PCR
IHC: Immunohistochemistry
TMA: Tissue microarray
ChIP: Chromatin-immunoprecipitation
IF: Immunofluorescence
SD: Standard deviation
