# Supplementary material for: ATAD2 interacts with C/EBPβ to promote esophageal squamous cell carcinoma metastasis via TGF-β1/Smad3 signaling
Source: J Exp Clin Cancer Res. 2021 Mar 23;40:109. doi: 10.1186/s13046-021-01905-x (PMC7986551; doi:10.1186/s13046-021-01905-x)
Supplement: Supplementary file 1 — Additional file 1: Table S1. Primers used for quantitative real-time PCR. [file 13046_2021_1905_MOESM1_ESM.docx]

Table S1. Primers used for quantitative real-time PCR

| **Target** | **Forward sequence** | **Reverse sequence** |
| --- | --- | --- |
| ATAD2 | 5′-TTGCTGGGTTATTTTAATCAT-3′ | 5′-ACTGTTTGACAAACCGCATCC-3′ |
| TGF-β1 | 5´- CGACTACTACGCCAAGGA -3´ | 5´-GAGAGCAACACGGGTTCA-3´ |
| C/EBPβ | 5′-TTTGTCCAAACCAACCGCAC-3′ | 5′-CCCCCAAAAGGCTTTGTAACC-3′ |
| Snail | 5′-TGGCAGCTTTTGTGAATCTG-3′ | 5′-AGGTGTTGGTTGCACCTTTC-3′ |
| E-cadherin | 5′-CAGTCTAGGCCAGTGCATCA-3′ | 5′-TTGCCCTCTGCTTTGTTCTT-3′ |
| N-cadherin | 5′-GGCAAGCAGAACTCCTCAAC-3′ | 5′-TCATTTAGTGCCGCTCTGTG-3′ |
| Vimentin | 5′-GAGAACTTTGCCGTTGAAGC-3′ | 5′-TCCAGCAGCTTCCTGTAGGT-3′ |
