# Supplementary material for: ATAD2 interacts with C/EBPβ to promote esophageal squamous cell carcinoma metastasis via TGF-β1/Smad3 signaling
Source: J Exp Clin Cancer Res. 2021 Mar 23;40:109. doi: 10.1186/s13046-021-01905-x (PMC7986551; doi:10.1186/s13046-021-01905-x)
Supplement: Supplementary file 2 — Additional file 2: Table S2. Univariate and multivariate analyses of overall survival in all patients. [file 13046_2021_1905_MOESM2_ESM.docx]

Table S2. Univariate and multivariate analyses of overall survival in all patients.

|  | **Univariate Analysis** | | | **Multivariate Analysis** | |
| --- | --- | --- | --- | --- | --- |
| **Variables** | **HR (95%CI)** | | ***P*** | **HR (95%CI)** | ***P*** |
| **All patients (n = 112)** | | | | | |
| **gender** |  | |  |  |  |
| Male | 0.90 (0.51-1.59) | | 0.713 | 1.08 (0.56-2.07) | 0.815 |
| Female |  | |  |  |  |
| **Age (years)** |  | |  |  |  |
| ≤ 55 | 1.24 (0.68-2.25) | | 0.484 | 1.34 (0.66-2.71) | 0.415 |
| > 55 |  | |  |  |  |
| **Clinical stage** |  | |  |  |  |
| I-II | 2.07 (1.19-3.6) | | **0.01** | 2.17 (1.16-4.05) | **0.015** |
| III-IV |  | |  |  |  |
| **Histologic Grade** |  | |  |  |  |
| G1 |  | |  |  |  |
| G2 | 2.28 (1.03-5.05) | | **0.042** | 1.51 (0.65-3.49) | 0.335 |
| G3 | 3.5 (1.53-8.04) | | **0.003** | 2.28 (0.92-5.65) | 0.077 |
| **Tumor size** |  | |  |  |  |
| ≤ 3cm | 1.44 (0.84-2.49) | | 0.186 | 1.96 (1.10-3.49) | **0.023** |
| > 3cm |  | |  |  |  |
| **ATAD2** |  | |  |  |  |
| Low | 1.63 (1.1-2.4) | | **0.015** | 2.44(1.36-4.37) | **0.003** |
| high |  |  | |  |  |

*P* < 0.05 in bold.
