# Supplementary material for: ATAD2 interacts with C/EBPβ to promote esophageal squamous cell carcinoma metastasis via TGF-β1/Smad3 signaling
Source: J Exp Clin Cancer Res. 2021 Mar 23;40:109. doi: 10.1186/s13046-021-01905-x (PMC7986551; doi:10.1186/s13046-021-01905-x)
Supplement: Supplementary file 3 — Additional file 3: Table S3. KEGG pathways of overlapping genes from DAVID analysis. [file 13046_2021_1905_MOESM3_ESM.docx]

Table S3. KEGG pathways of overlapping genes from DAVID analysis.

| **Term** | ***P*** | **Genes** |
| --- | --- | --- |
| hsa04350:TGF-beta signaling pathway | **0.005** | INHBA, TGFB3, SMAD3, THBS1, CHRD, BMP6 |
| hsa04360:Axon guidance | **0.007** | ABLIM2, EPHA7, CXCR4, SEMA3D, NTNG2, L1CAM, EPHA2 |
| hsa04068:FoxO signaling pathway | **0.008** | IRS2, PIK3CD, TGFB3, SMAD3, GADD45B, HOMER2, AGAP2 |
| hsa04115:p53 signaling pathway | **0.012** | CCNE2, TP53I3, PMAIP1, THBS1, GADD45B |
| hsa04978:Mineral absorption | **0.020** | TRPM6, ATP1B1, ATP1A3, SLC40A1 |
| hsa04923:Regulation of lipolysis in adipocytes | **0.038** | CGA, IRS2, PTGS2, PIK3CD |
| hsa04710:Circadian rhythm | 0.063 | NR1D1, PER1, BHLHE40 |
| hsa04110:Cell cycle | 0.081 | CDKN1C, CCNE2, TGFB3, SMAD3, GADD45B |
| hsa04960:Aldosterone-regulated sodium reabsorption | 0.094 | ATP1B1, PIK3CD, ATP1A3 |

*P* < 0.05 in bold
