# Supplementary figures and images for: ATAD2 interacts with C/EBPβ to promote esophageal squamous cell carcinoma metastasis via TGF-β1/Smad3 signaling
Source: J Exp Clin Cancer Res. 2021 Mar 23;40:109. doi: 10.1186/s13046-021-01905-x (PMC7986551; doi:10.1186/s13046-021-01905-x)

Figure S1


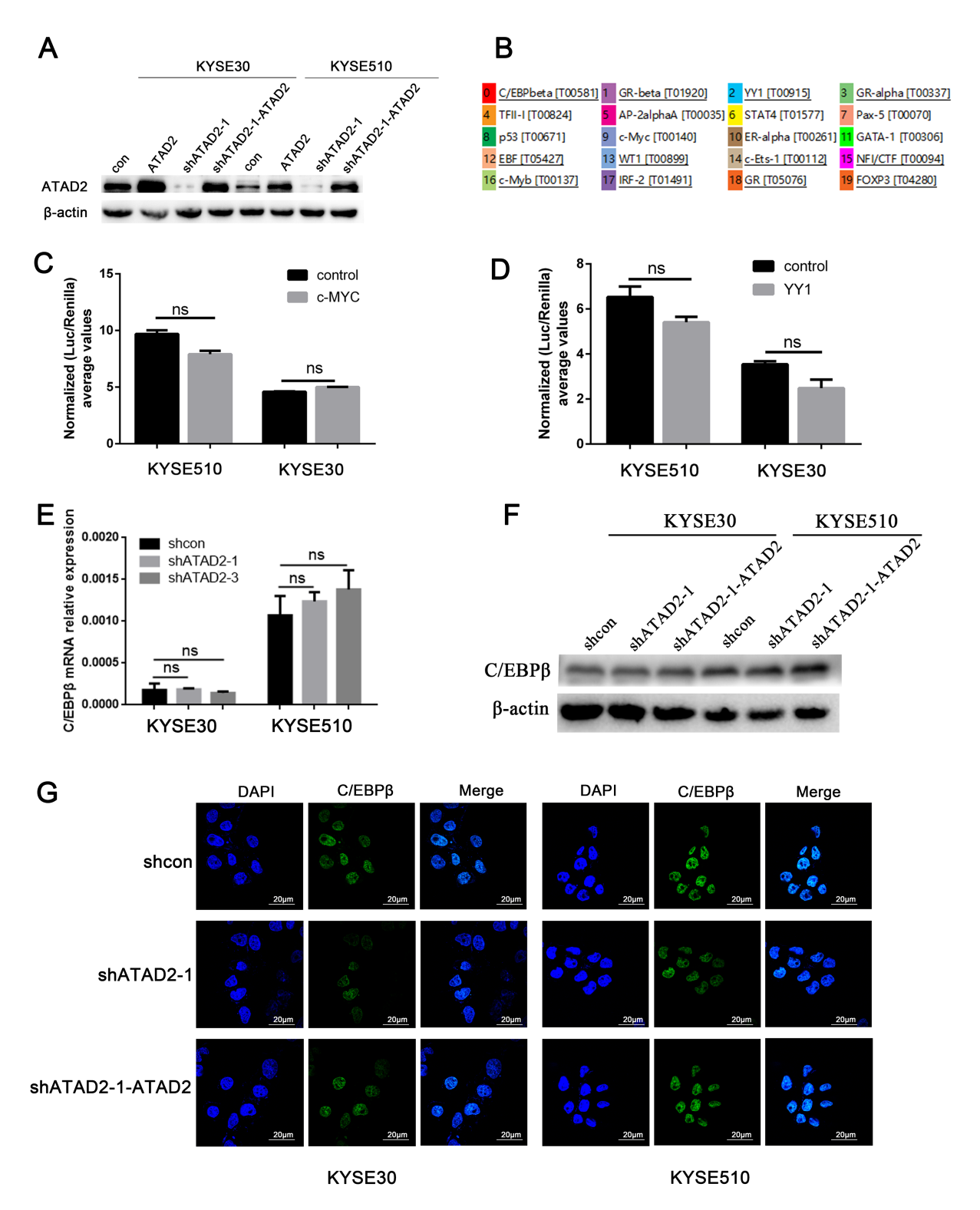

Supplement: Supplementary file 4 — Additional file 4: Supplemental Figure 1. (A) ATAD2 overexpression, knockdown, and restoration in KYSE30 and KYSE510 cells were confirmed by western blot analysis. (B) Predicted transcription factors of TGF-β1 gene obtained from PROMO dataset. (C, D) TGF-β1 luciferase activity induced by YY1 (C) and c-MYC (D) in KYSE510 and KYSE30 cells. (E, F) Effect of ATAD2 knockdown on C/EBPβ mRNA (E) and protein expression (F) in KYSE30 and KYSE510 cells were validated by qRT-PCR and western blot respectively. (G) Effects of ATAD2 knockdown and restoration on localization of C/EBPβ (green) in KYSE30 and KYSE510 cells performed by Immunofluorescence staining. Data was presented as mean ± SD. (ns: non-significant difference was detected). [file 13046_2021_1905_MOESM4_ESM.docx]
